# The translational study of apathy—an ecological approach

**DOI:** 10.3389/fnbeh.2015.00241

**Published:** 2015-09-09

**Authors:** Flurin Cathomas, Matthias N. Hartmann, Erich Seifritz, Christopher R. Pryce, Stefan Kaiser

**Affiliations:** ^1^Preclinical Laboratory for Translational Research into Affective Disorders (PLaTRAD), Department of Psychiatry, Psychotherapy and Psychosomatics, Psychiatric Hospital, University of ZurichZurich, Switzerland; ^2^Department of Psychiatry, Psychotherapy and Psychosomatics, Psychiatric Hospital, University of ZurichZurich, Switzerland; ^3^Laboratory for Social and Neural Systems Research, Department of Economics, University of ZurichZurich, Switzerland; ^4^Neuroscience Center, Swiss Federal Institute of Technology, University of ZurichZurich, Switzerland; ^5^Zurich Center for Integrative Human Physiology, University of ZurichZurich, Switzerland

**Keywords:** apathy, schizophrenia, depression, psychopathology, ecological animal models

## Abstract

Apathy, a quantitative reduction in goal-directed behavior, is a prevalent symptom dimension with a negative impact on functional outcome in various neuropsychiatric disorders including schizophrenia and depression. The aim of this review is to show that interview-based assessment of apathy in humans and observation of spontaneous rodent behavior in an ecological setting can serve as an important complementary approach to already existing task-based assessment, to study and understand the neurobiological bases of apathy. We first discuss the paucity of current translational approaches regarding animal equivalents of psychopathological assessment of apathy. We then present the existing evaluation scales for the assessment of apathy in humans and propose five sub-domains of apathy, namely self-care, social interaction, exploration, work/education and recreation. Each of the items in apathy evaluation scales can be assigned to one of these sub-domains. We then show that corresponding, well-validated behavioral readouts exist for rodents and that, indeed, three of the five human apathy sub-domains have a rodent equivalent. In conclusion, the translational ecological study of apathy in humans and rodents is possible and will constitute an important approach to increase the understanding of the neurobiological bases of apathy and the development of novel treatments.

## Introduction

Apathy has initially been defined as a lack of motivation (Marin, 1990). Recently, a more behavioral definition has been introduced, defining apathy as a quantitative reduction in goal-directed behavior (Levy and Dubois, 2006). In the present review this latter definition, avoiding the psychological term motivation, will be used because it is much more compatible with the translational approach. Apathy is a prevalent symptom dimension in psychiatric and neurologic disorders (Marin, 1990; Levy and Dubois, 2006; Foussias and Remington, 2010). In psychiatry, it can be observed in schizophrenia (Blanchard and Cohen, 2006), major depression (Marin et al., 1993), and as a consequence of drug abuse (Lynskey and Hall, 2000). In neurological disorders, apathy has been observed independently of other psychiatric symptoms; for example in basal ganglia disease (Levy and Czernecki, 2006; Starkstein et al., 2009), Alzheimer’s disease (Starkstein et al., 2001; Robert et al., 2010) and stroke (Jorge et al., 2010; Caeiro et al., 2013). Although questions have been raised concerning the nosological status of apathy (Starkstein and Leentjens, 2008), there is unequivocal evidence for its negative impact on the functional outcome of the above mentioned disorders (Kiang et al., 2003; van Reekum et al., 2005; Faerden et al., 2009; Foussias et al., 2009; Konstantakopoulos et al., 2011). Additionally, with the exception of some promising trials with methylphenidate to treat apathy observed in neurological disorders (Devos et al., 2013; Rea et al., 2014), the treatment of apathy remains a rather neglected area of therapeutic research.

Apathy is a symptom dimension that cuts across disease categories. In the present review it is therefore proposed that research into the neurobiological bases and treatment of apathy is best served by regarding it as a domain that is, to a large extent, applicable across traditional nosological categories. In other words, apathy can be considered as a trans-diagnostic clinical phenotype. In addition to, and indeed facilitated by, the conception of apathy as a domain, we propose that understanding of apathy can be increased by integrating its study in human patients with studies in animal models. In contrast to symptoms/domains that have been studied extensively in animal models, with examples including anxiety and helplessness, apathy has become a focus of animal studies only relatively recently, and there has been an almost exclusive focus on discrete behavioral tests rather than the assessment of spontaneous behavior. Based on these considerations in the present review, we: (1) note the current deficits in translational approaches to the study of apathy in the ecological setting; (2) propose five sub-domains of apathy that can be measured by psychopathological examination in humans; and (3) demonstrate how assessment of specific animal behaviors can yield analogs for some of these human apathy sub-domains. In other words, we complement existing translational discrete task-based approaches by translating human psychopathological assessments into behavioral observation of spontaneous, on-going animal behavior.

## Translating Apathy from Humans to Animal Models

There has been considerable debate about the challenges of translating progress in behavioral neuroscience to the clinic (Markou et al., 2009; Pryce and Seifritz, 2011; Machado-Vieira, 2012; Braff and Braff, 2013). Consensus is emerging that drug research and development should not be aimed at treating entire categorical disease entities as currently conceptualized, but rather at treating specific symptom dimensions, including those that pertain across neuropsychiatric disorders. As a consequence, translational models comprising manipulations and readout assays that provide animal equivalents of the targeted domains are needed (Markou et al., 2009; Pratt et al., 2012). Indeed, given that it is more realistic to develop an animal model for a specific domain than for an entire nosological disorder, the domain concept also brings the advantage of being contiguous with the translational approach.

**Figure 1:**
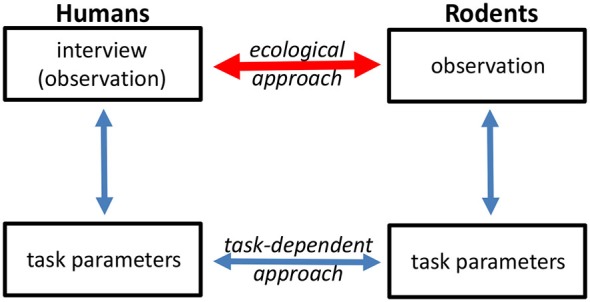
**Different approaches can be used to translate human symptoms into animal behavior readouts, and *vice versa***. One approach (task-dependent) is to assess behavior at the level of discrete task performance. The ecological approach aims to link human psychopathological assessment with observation of spontaneous animal behavior.

Driven largely by its impact on functional outcome and resistance to current treatments, apathy is becoming an increasing focus of psychiatric research (Simpson et al., 2012; Foussias et al., 2015). Interview-based rating scales remain the gold standard in the clinical assessment of apathy. Obviously, a direct translation to animal models is not possible. However, the information obtained through interview-based assessments can, at least in part, be assessed through the observation of spontaneous animal behavior. This approach will be the focus of the present review. Another way used to circumvent these challenges of translational research has involved the establishment of discrete neuropsychological tasks in humans and of equivalent tasks in various animal models (Figure 1). For example, several groups have applied tasks to assess effort-based decision-making in humans and animals as a behavioral proxy for motivational deficits (e.g., Gold et al., 2013; Fervaha et al., 2013; Markou et al., 2013; Hartmann et al., 2014; Salamone et al., 2015).

However, pursuing this approach is not straightforward and comprises a number of steps in each species, each of which poses its own challenges and pitfalls. The first step is the development of appropriate tasks to assess apathy in humans. While tasks have been considered to be “objective” and thus preferable, interview-based assessment with rating scales remain the gold standard in the assessment of apathy and the measure against which all newly developed tasks are tested. The second step concerns the development of tasks that are analogs in humans and model species with respect to the neurobehavioral processes they are measuring. The third step is the animal model equivalent of step one described above for humans, namely, interpretation of task performance in terms of what it implies about their spontaneous, on-going behavior in the home-cage. Does an animal that exhibit low goal-directed behavior in a discrete task also exhibit decreased performance of spontaneous behaviors in its home environment? Although one could argue that task behavior in itself is enough, validity of the ecological approach would be markedly increased if task performance had similar relationships to “real-world” behavior, both in humans and animal models.

In the present review, we propose an ecological approach, as an important complement to discrete tasks, to the development of translational assays for apathy, based on linking human psychopathological assessment and the observation of spontaneous animal behavior. We propose five sub-domains of apathy, some of which can be observed and quantified in laboratory model animals.

## Sub-Domains of Apathy in Humans

In this section, we will briefly introduce approaches to the assessment of apathy in humans. As mentioned above, interview-based rating scales remain the gold standard for the assessment of apathy, but have been complemented by self-ratings and observer-ratings. Based on the items used to assess apathy, we propose a five sub-domain schema.

Scales aiming at the exclusive assessment of apathy have mainly been developed for patients with neurological disorders, such as the Apathy Evaluation Scale (AES; Marin et al., 1991). However, the AES has also been used to assess apathy in schizophrenia and depression (Faerden et al., 2008; Raskin et al., 2012). While the scale items themselves do not specify sub-domains of apathy, the accompanying manual suggests some domains for assessing interest and activities, e.g., hobbies, work or activities with other people.

In patients with primary psychiatric disorders apathy is usually addressed in the context of a broader negative symptom assessment, such as the Scale for the Assessment of Negative Symptoms (SANS; Andreasen, 1989). Factor analytic studies have demonstrated that these instruments assess two different factors—apathy/avolition and diminished expression (Messinger et al., 2011). The Brief Negative Symptom Scale (BNSS; Kirkpatrick et al., 2011) and the Clinical Assessment Interview for Negative Symptoms (CAINS; Kring et al., 2013) are two recent instruments that reflect this two-factor structure even more clearly. In all these instruments the assessment of apathy relies on the elicitation of reports on activities in the domains work, recreation, social/sexual interest, exploration and self-care.

While interview-based scales remain the standard for assessment of apathy in humans, there are some inherent limitations. For example, the retrospective reporting by the patient regarding his activities and mental state during a certain time frame might lack reliability. To circumvent this problem, observer-based measures have been employed. For the AES a carer version is available that can be completed by professional and non-professional caregivers (Marin et al., 1991). For inpatient settings, Trémeau et al. (2012) have introduced a measure based on constant observation. Importantly, all these observation-based measures address the same sub-domains of apathy as the interview-based scales, although opportunities for activities are more limited in inpatient settings. In addition, newer technologies facilitate real-time assessment of patients’ behavior and associated internal states (Oorschot et al., 2009), but have not yet been applied to the study of apathy.

A primary goal of current rating scales to assess apathy is the estimation of goal-directed behavior in a wide array of life domains. Based on the domains assessed in interview- and observer-based rating scales, we propose five sub-domains in which a reduction of goal-directed behavior is frequently observed in apathetic patients, namely: self-care, exploration, social interaction, work/education, and recreation.

## Apathy in Laboratory Animals

In the current section, we discuss the potential of applying observational measurement of spontaneous behavior in mice to the translational study of apathy. The different behaviors identified are considered in the framework of the five sub-domains of apathy in humans (Table 1). For each behavior, an example of a manipulation that impacted on the behavior to induce changes relevant to the corresponding sub-domain, is presented. The discussion is limited to rodents, but should certainly be extended to other species studied in the laboratory.

**Table 1:** **Assignment of the different human and animal psychopathological assessments/observations to the different sub-domains of apathy**.

	Human	Rodent
Self-care	Neglected personal hygiene	Impaired nest construction
	Neglected clothing	Disturbed self-grooming
	Reduced care for place to live
Social interaction	Reduced participation in activities with other people	Reduced maternal care
	Reduced discussion of personal matters with others	Reduced interest in same and opposite sex-conspecifics
	Reduced sexual interest and activity
Exploration	Impaired novelty seeking	Impaired interest in novel objects
Recreation	Reduced interest and engagement in recreational activities	–
Work/Education	Reduced interest and activity in work/education	–

### Nest Construction

Self-care is an important sub-domain of apathy in humans and includes activities related to the patient’s home, such as cleaning or looking for a place to live (Table 1). Nest construction is common across many animal species and is important for heat conservation, reproduction and as a protective place against predators (Latham and Mason, 2004). Several scales with which to rate nest construction have been proposed. A comprehensive scale was created by Deacon (Deacon, 2006) consisting of a five-point rating scale, aimed at assessing the quality of the nest.

It was shown that the sub-chronic administration of phencyclidine, which is well-characterized as a pharmacological inducer of schizophrenia-relevant behaviors in animals, impaired nest building in mice (Pedersen et al., 2014). The deficit was quantified by a scoring system (“Nesting Index Score”), where the quality of the nest was assessed every hour. The deficit was attenuated by acute treatment with a nicotine alpha7 receptor agonist (the same compound has been reported to exert beneficial effects on negative symptoms in schizophrenia patients; Hashimoto, 2015), but not with risperidone, an atypical antipsychotic.

### Self-Grooming

Another aspect of self-care in humans includes personal hygiene, which is often impaired in patients with apathy (Table 1). The rodent equivalent is self-grooming and accounts for 30–50% of the waking time of rodents (Bolles, 1960). The main function of self-grooming is to clean in order to maintain health, e.g., by removing detritus and disease-carrying parasites. Other functions of self-grooming include thermoregulation, stimulation of pheromone release and self-stimulation (Spruijt et al., 1992). In rodents, self-grooming consists of a repeated behavior with a sequential cephalocaudal progression (Kalueff and Tuohimaa, 2004). The usual method for measuring self-grooming is by observation and quantification of duration, frequency and inter-bout intervals of self-grooming elements (Kalueff and Tuohimaa, 2004). In addition, complex algorithms exist for detailed descriptions of repeated self-grooming sequences using a computer-assisted scoring system (Kalueff et al., 2007).

Denmark et al. (2010) showed that chronic social defeat stress (CSDS) leads to disorganized patterning of grooming behaviors in subordinate mice. CSDS is a widely applied manipulation in rodent research and it is based on the finding that specific uncontrollable stressful life events are major aetiological factors in various neuropsychiatric disorders. It was shown that CSDS affects dopamine function in specific brain regions, e.g., down-regulated expression of genes for dopamine receptors and signaling proteins (Azzinnari et al., 2014).

### Interest in Same and Opposite-Sex Conspecifics

Social withdrawal or asociality is a key component of apathy in humans that includes interaction with family and friends as well as sexual interest and activity (Table 1). Rodents exhibit a complex repertoire of social behaviors (Grant and Mackintosh, 1963). One of the methods to test interest in same-sex conspecifics in mice is the three-chambered social approach test, initially developed as a discrete test, it can also be applied in the home-cage setting (Yang et al., 2011): the home-cage is separated into three parts and the rodent can move from one part to another. In one outer compartment there is a wire cage containing an unfamiliar conspecific and in the opposite outer compartment there is an empty wire cage (Nadler et al., 2004). Measures that can be obtained include time spent in the compartment containing the novel subject compared to time in the empty compartment (Yang et al., 2011).

A large number of behaviors can be measured to assess socio-sexual and copulatory behavior: sniffing (e.g., total duration of anogentital sniffing), number of male mounts (defined as male using both forepaws to climb onto a female from behind for copulation but without achieving penetration), female lordosis (all four paws rounded, the hind region elevated from the floor), intromission (two or more thrusts with penetration) and ejaculation (Huber et al., 1980; Farmer et al., 2014).

The bilateral depletion of dopamine in the medial prefrontal cortex (mPFC) through infusion of 6-hydroxydopamine (6-OHDA) in adult and adolescent rats led to reduced social interactions (e.g., sniffing time) compared to controls (Li et al., 2010). The 6-OHDA-mPFC lesion model of dopaminergic neurons is based on the findings that there is a correlation between hypodopaminergic functioning in the PFC and negative symptoms (Howes and Kapur, 2009).

### Maternal Care

Another aspect of social interaction is maternal care, which is not included in routine assessment of apathy. However, apathetic behavior towards newborn infants is an important issue in mothers suffering from schizophrenia and other psychiatric disorders (Matevosyan, 2011). Also in rodents, maternal care—the nurturant behavior of the dam towards its offspring—is one of the most complex social relationships (Lonstein and De Vries, 2000). It consists of different behaviors that can be separately assessed (Lonstein and Fleming, 2002; Kuroda and Tsuneoka, 2013): (A) Retrieval: the mother picking up a pup in its mouth and transporting it to the nest; (B) Pup licking: the functions of pup licking are to clean, increase activity and facilitate elimination of waste; and (C) Time spent crouching over and nursing pups: the mother is positioned over the pups and is either active (licking the pups, nursing, self-grooming, moving nest material) or quiescent (induced by the suckling of the pups).

Examples of effects of schizophrenia-relevant manipulations on maternal care include a study in mice where maternal behavior (e.g., the latency to retrieve pups) was impaired by genetic deletion of the endocannabinoid receptor-1 (Schechter et al., 2013). In human patients, long-term cannabis use is associated with apathy (Looby and Earleywine, 2007) and an imaging study identified an inverse relationship between striatal dopamine synthesis capacity and apathy (measured with the AES; Bloomfield et al., 2014).

### Novel Object Exploration Test

Exploration can be defined as behavior that is elicited by novel stimuli and allows for the collection of information about unfamiliar elements of the environment (Crusio and van Abeelen, 1986). The reduction of interest in novel experiences, including attenuated exploration, is an indicator of human apathy (Table 1). In rodents, novelty is arousing and stimulates exploration. Whenever a novel stimulus is presented alongside a familiar one, there is typically more exploration of the novel stimulus until the stimulus loses its novelty (Ennaceur, 2010). There are different modifications of the novel object exploration (or preference) test based on the same principle: Rodents are exposed to both familiar and novel objects (e.g., in their home-cages) and allowed to explore them freely (Pearson et al., 2011; Blick et al., 2014). The interaction time of the subject with the novel object is measured and compared to the time spent with the familiar object (Heyser and Chemero, 2012).

It was shown in mice that a compound with selective partial agonist effects to the dopamine D4 receptor and minimal affinity for dopamine D2 and D3 receptors increased time spent near a novel object compared to controls (Powell et al., 2003).

## Conclusion and Implications for Future Research

Apathy, which is defined in the present review as a quantitative reduction in goal-directed behavior, is a prevalent symptom dimension that cuts across disease categories and has a negative impact on the functional outcome of many neuropsychiatric disorders including schizophrenia and depression. Translational studies in humans and animal models are an important approach to disentangle the underlying neurobiological mechanisms. Recently, considerable effort has been made in the assessment of apathy via task performances (e.g., effort-based decision-making). While this approach is indeed interesting and promising, the assessment of apathy in humans through rating scales remains the gold standard. We therefore propose a complementary approach by using the clinical interviews as a starting point and show that the items can be synthesized into five sub-domains in humans and that three of these can also be assessed in rodents.

Apathy is clearly not unique in being conducive to this approach. Another example would be sleep disturbance, as is common in many neuropsychiatric disorders, for which there are several detailed human assessment scales (e.g., the Pittsburgh Sleep Quality Index; Buysse et al., 1989) as well as methods of quantification in animal models (e.g., home-cage monitoring systems; Cathomas et al., 2015) available. In conclusion, we propose that future efforts of translational assessment of apathy in humans and animal models should include behavioral readouts based on interview/observational measures, preferably conducted in the real-life/home-cage. The next step in the development of this approach will be to demonstrate that the readouts in humans and animals are sensitive to the same manipulation. For example, administration of a dopamine-receptor modulating compound could be investigated for effects on the proposed sub-domains of apathy, e.g., assessing self-care in humans using the SANS and self-grooming in mice. This approach, in combination with task-based assessments, should lead to improved face validity of animal apathy models and can then be applied to assess the effects of relevant genetic, environmental and pharmacological manipulations to increase understanding of the neurobiological bases of apathy and for the development of new treatments.

## Conflict of Interest Statement

The authors declare that the research was conducted in the absence of any commercial or financial relationships that could be construed as a potential conflict of interest.
